# Supplementary material for: Myosin forces remodel F-actin for mechanosensitive protein recognition
Source: Nature. 2026 Apr 22;654(8117):240–9. doi: 10.1038/s41586-026-10398-7 (PMC13233326; doi:10.1038/s41586-026-10398-7)
Supplement: Supplementary file 1 — This file contains a Supplementary Discussion, which features additional analysis of coarse-grained molecular dynamics simulations focused on the effects of torque, the temporal evolution of elliptical supercoil cross-sections, and how perturbations of F-actin’s mechanical properties and helical architecture impact supercoil properties. [file 41586_2026_10398_MOESM1_ESM.pdf]

nature portfolio

https://doi.org/10.1038/s41586-026-10398-7

Supplementary information

Myosin forces remodel F-actin for mechanosensitive protein recognition

In the format provided by the authors and unedited

Supplementary Discussion

Torque does not produce supercoils

While myosin motors primarily exert axial forces along actin filaments, biophysical studies have also suggested that they apply modest torques[57,58]. To assess the contribution of this force regime to supercoiling, we performed simulations with two magnitudes of torques (80 pN and 800 pN), in both over- and under-twisting directions in the absence and presence of bond breaking (Extended Data Fig. 4e; Supplementary Video 6). In all cases, torque resulted in immediate helical twist remodeling of the filament. With bond breaking enabled, both directions and force magnitudes resulted in rapid filament rupture without the formation of supercoils. With bond breaking disabled, at the lower torque magnitude the filament retained a straight morphology despite internal twist remodeling. At the higher torque magnitude, it underwent a physically implausible looping transition morphologically distinct from the supercoils we observe experimentally. Collectively, these analyses suggest axial compression generated by stochastically operating motor ensembles can produce F-actin supercoils, regardless of motor directionality, without the requirement for torque.

F-actin properties specify supercoil architecture

We next explored how F-actin's helical lattice architecture and mechanical properties impact the morphology of supercoils. For computational efficiency, these simulations were performed with a uniform constant compressive force (6 pN) on a 200-subunit filament with bond breaking disabled. We first systematically varied F-actin's helical twist and rise. While varying twist modestly but significantly impacted supercoil pitch (Extended Data Fig. 3g), it had no significant effect on supercoil peak-to-peak amplitude. Conversely, both supercoil amplitude and pitch display a significant linear positive relationship with helical rise (Extended Data Fig. 3g). To assess the influence of F-actin's mechanical properties, we focused on persistence length as a proxy for flexural rigidity (Methods). Persistence length also impacted supercoil pitch, with more rigid filaments displaying significantly longer pitch, without a significant effect on supercoil amplitude (Extended Data Fig. 3h). These simulations collectively suggest F-actin's architecture and mechanics both have the capacity to modulate supercoiling, with F-actin's helical rise uniquely

determining the magnitude of supercoil peak-to-peak amplitude, the key parameter for establishing a supercoiled F-actin region.

As we observed in our *in vitro* cryo-ET experiments (Fig. 2b,c), supercoils featured elliptical cross-sections in simulations (Fig. 2e; Extended Data Fig. 4c). While modulating F-actin's helical parameters and persistence length did impact supercoil cross-section eccentricity (Extended Data Fig. 3i), no clear trends are apparent, and a high degree of ellipticity persists in all conditions we examined. Plotting ellipticity versus simulation frame (Extended Data Fig. 4h) in the continuous compression condition revealed maximal ellipticity at the beginning of each trajectory, which either remained high or decreased as individual supercoils evolved. Consistently, a segment which will supercoil initially undergoes uniplanar buckling, subsequently supercoiling as the end-to-end distance of the filament continues to decrease, resulting in an elliptical cross-section (Supplementary Video 6). Overall, these simulations suggest that the morphology of supercoils are broadly linked to F-actin's structure and mechanics, while their asymmetric cross-sections are the product of the dynamics leading to their formation in the presence of compressive forces.
