# Supplementary material for: Myosin forces remodel F-actin for mechanosensitive protein recognition
Source: Nature. 2026 Apr 22;654(8117):240–9. doi: 10.1038/s41586-026-10398-7 (PMC13233326; doi:10.1038/s41586-026-10398-7)
Supplement: Supplementary file 2 — Reporting Summary [file 41586_2026_10398_MOESM2_ESM.pdf]

nature portfolio

Corresponding author(s): Gregory Alushin

Last updated by author(s): Dec 30, 2025

Reporting Summary

Nature Portfolio wishes to improve the reproducibility of the work that we publish. This form provides structure for consistency and transparency in reporting. For further information on Nature Portfolio policies, see our Editorial Policies and the Editorial Policy Checklist.

Statistics

For all statistical analyses, confirm that the following items are present in the figure legend, table legend, main text, or Methods section.

n/a	Confirmed	
☐	☒	The exact sample size (*n*) for each experimental group/condition, given as a discrete number and unit of measurement
☐	☒	A statement on whether measurements were taken from distinct samples or whether the same sample was measured repeatedly
☐	☒	The statistical test(s) used AND whether they are one- or two-sided<br>*Only common tests should be described solely by name; describe more complex techniques in the Methods section.*
☒	☐	A description of all covariates tested
☐	☒	A description of any assumptions or corrections, such as tests of normality and adjustment for multiple comparisons
☐	☒	A full description of the statistical parameters including central tendency (e.g. means) or other basic estimates (e.g. regression coefficient) AND variation (e.g. standard deviation) or associated estimates of uncertainty (e.g. confidence intervals)
☐	☒	For null hypothesis testing, the test statistic (e.g. *F*, *t*, *r*) with confidence intervals, effect sizes, degrees of freedom and *P* value noted<br>*Give P values as exact values whenever suitable.*
☒	☐	For Bayesian analysis, information on the choice of priors and Markov chain Monte Carlo settings
☒	☐	For hierarchical and complex designs, identification of the appropriate level for tests and full reporting of outcomes
☒	☐	Estimates of effect sizes (e.g. Cohen's *d*, Pearson's *r*), indicating how they were calculated

Our web collection on statistics for biologists contains articles on many of the points above.

Software and code

Policy information about availability of computer code

Data collection	Cryo-EM and Cryo-ET data were collected with SerialEM. Low-magnification cryo-EM data (displayed in Figure 1) were collected with Leginon. Epi-fluorescence movies were collected with MetaMorph (Molecular Dynamics). TIRF images were collected with Nikon NIS-Elements. Cryo-fluorescence images were collected with Leica's LAS X software.
Data analysis	For cellular cryo-ET analysis, direct detector frame series were aligned with MotionCor2, and CTF estimation was performed with CTFFIND4. Tilt series were aligned with AreTomo2, then reconstructed with IMOD. Reconstructed tomograms were denoised with cryoCARE and IsoNET. Segmentation was performed with Dragonfly and MemBrain v2.<br><br>For in vitro cryo-ET analysis, frames were aligned with MotionCor2. Tilt series were aligned with Appion-Protomo, then reconstructed with Tomo3D. Tomograms were denoised with a custom denoising autoencoder approach, implemented with TensorFlow and functions from the EMAN2 package in Python.<br><br>For cryo-EM analysis, direct detector frame series were aligned with MotionCor2, and CTF estimation was performed with CTFFIND4. Filament picking and curvature analysis were performed with a custom denoising autoencoder approach, implemented with TensorFlow and functions from the EMAN2 package in Python. Classification, reconstruction, and variability analysis were performed with cryoSPARC v3.2.0 and RELION v3.1.2. Molecular dynamics flexible fitting was performed with ISOLDE on atomic models idealized with Phenix.<br><br>Coarse-grained molecular dynamics simulations were performed with ESPResSO and analyzed with custom code in Python.<br><br>Fluorescence microscopy data were analyzed with FIJI. UCSF Chimera and ChimeraX were used for molecular graphics and structural analysis. Statistical analysis and plotting were conducted with GraphPad Prism, SciPy, and Matplotlib.

Custom code was prepared with the assistance of ChatGPT 4.0.

All custom code is available at www.github.com/alushinlab/squiggle.

For manuscripts utilizing custom algorithms or software that are central to the research but not yet described in published literature, software must be made available to editors and reviewers. We strongly encourage code deposition in a community repository (e.g. GitHub). See the Nature Portfolio guidelines for submitting code & software for further information.

Data

Policy information about availability of data

All manuscripts must include a data availability statement. This statement should provide the following information, where applicable:

- Accession codes, unique identifiers, or web links for publicly available datasets
- A description of any restrictions on data availability
- For clinical datasets or third party data, please ensure that the statement adheres to our policy

Cryo-EM density maps have been deposited in the EMDB with the following accession codes: Myosin force-evoked supercoil F-actin (EMD-46426); Control myosin tethered F-actin -ATP 1 (EMD-46427); Control myosin tethered F-actin -ATP 2 (EMD-46428); Consensus force-activated α-catenin–F-actin complex (EMD-46429); 3DVA sorted force-activated α-catenin–F-actin complex (EMD-46431). Additional raw and processed data files are available at Zenodo (refs. 114,115,116). Cellular tomograms, segmentations, and filament traces are available at https://doi.org/10.5281/zenodo.18022754. Trained neural networks and denoised in vitro tomograms with filament traces are available at https://doi.org/10.5281/zenodo.12702199. Trained neural networks used for single particle analysis particle picking, flexibly fit PDB models, and variability analysis maps and models used for data analysis are available at https://doi.org/10.5281/zenodo.12702799. Source data are provided for Figs. 1-4 and Extended Data Figs. 1, 2, 6, and 10. Additional source data are available at Zenodo (refs. 117,118) for simulations (Fig. 2f-h and Extended Data Fig. 3; https://doi.org/10.5281/zenodo.17932417) and Extended Data Fig. 5 (https://doi.org/10.5281/zenodo.17958929). All reagents and resources reported in this study are freely available from the corresponding author.

Research involving human participants, their data, or biological material

Policy information about studies with human participants or human data. See also policy information about sex, gender (identity/presentation), and sexual orientation and race, ethnicity and racism.

Reporting on sex and gender	N/A
Reporting on race, ethnicity, or other socially relevant groupings	N/A
Population characteristics	N/A
Recruitment	N/A
Ethics oversight	N/A

Note that full information on the approval of the study protocol must also be provided in the manuscript.

Field-specific reporting

Please select the one below that is the best fit for your research. If you are not sure, read the appropriate sections before making your selection.

☒ Life sciences ☐ Behavioural & social sciences ☐ Ecological, evolutionary & environmental sciences

For a reference copy of the document with all sections, see nature.com/documents/nr-reporting-summary-flat.pdf

Life sciences study design

All studies must disclose on these points even when the disclosure is negative.

Sample size	Sample sizes were not pre-determined. The amount of data collected were limited by our capacity to prepare high-quality specimens and the length of cryo-EM imaging sessions. Combining multiple datasets only modestly enhanced the resolution of our reconstructions, suggesting that we had reached the limitations imposed by the molecular heterogeneity of our samples.
Data exclusions	No data were excluded from analysis.
Replication	F-actin sinusoidal regions were observed in three independent low magnification cryo-EM experiments (dual motor, pointed-end directed force, and barbed-end directed force conditions), three high-magnification single particle cryo-EM datasets in the dual motor condition, and two in vitro cryo-ET datasets (pointed-end directed force and barbed-end directed force). Imaging with both cryo-ET and cryo-EM visualized highly similar morphological features.
Randomization	For Fourier Shell Correlation based resolution analysis, particles were randomly assigned to half-datasets for comparison. For other

Randomization	experiments in this study, as is customary we did not perform randomization, since biophysical experiments are performed under highly controlled experimental conditions where co-variates are minimal. Furthermore, specific individual cells and molecules cannot be randomly assigned to groups: instead, large numbers of molecules or cells from a population are subdivided into experimental conditions (e.g. aliquots from a tube) for comparison. As these divisions are not controlled, they effectively achieve a similar effect as intentional randomization of individual subjects.
Blinding	To avoid bias in manual analysis of cellular tomograms, blinding was performed for analysis presented in Fig. 1C / Extended Data Fig. 1f, as described in the Methods. Different investigators performed experiments and analysis; thus, those performing analysis were blinded to group allocation during data collection.<br><br>For other comparisons between experimental conditions (e.g. reconstitution experiments -/+ ATP), blinding was not performed. Analysis was performed with automated methods with minimal human intervention for these experiments, and thus blinding was not required.

Reporting for specific materials, systems and methods

We require information from authors about some types of materials, experimental systems and methods used in many studies. Here, indicate whether each material, system or method listed is relevant to your study. If you are not sure if a list item applies to your research, read the appropriate section before selecting a response.

Materials & experimental systems

n/a	Involved in the study
☐	☒ Antibodies
☐	☒ Eukaryotic cell lines
☒	☐ Palaeontology and archaeology
☒	☐ Animals and other organisms
☒	☐ Clinical data
☒	☐ Dual use research of concern
☒	☐ Plants

Methods

n/a	Involved in the study
☒	☐ ChIP-seq
☒	☐ Flow cytometry
☒	☐ MRI-based neuroimaging

Antibodies

Antibodies used	We used monoclonal anti-GFP antibody G6539, purchased from Sigma-Aldrich, to anchor GFP-tagged myosin motor proteins to the surface of cryo-EM grids.
Validation	This is a widely-used commercial anti-GFP antibody, which according to the manufacturer has been cited in 170 publications. The manufacturer provides a certificate of analysis for each batch of the antibody, validated through its performance in western-blotting with appropriate controls. Given its widespread use, we did not independently validate the antibody, beyond its apparent success in anchoring myosin to grids in a manner which maintains motility (evident in Supplementary Video 2).

Eukaryotic cell lines

Policy information about cell lines and Sex and Gender in Research

Cell line source(s)	PtK2 (#CCL-56) and HEK293T cells (#CRL-3216) were purchased from ATCC.
Authentication	ATCC validates cell lines by STR profiling. We did not further validate lines after purchase.
Mycoplasma contamination	Cells were monitored for mycoplasma contamination with the ATCC universal mycoplasma detection kit (a PCR-based assay), and tested negative.
Commonly misidentified lines (See ICLAC register)	No commonly mis-identified cell lines were used in this study.

Plants

Seed stocks	N/A
Novel plant genotypes	N/A
Authentication	N/A
